# Metastatic Neuroblastoma Mimicking Multiple Infantile Hemangiomas Diagnosed With Ultrasound

**DOI:** 10.7759/cureus.73013

**Published:** 2024-11-04

**Authors:** Ashley N Houff, Sharon E Albers, Monique Kumar

**Affiliations:** 1 Dermatology, College of Medicine, University of Central Florida, Orlando, USA; 2 Dermatology, Morsani College of Medicine, University of South Florida, Tampa, USA; 3 Dermatology, AdventHealth, Orlando, USA

**Keywords:** dermatology, infant, infantile neuroblastoma, pediatric dermatology, skin neoplasms, ultrasonography

## Abstract

Neuroblastoma is a malignant tumor derived from the neural crest cells that often involves the adrenal glands and rarely metastasizes to the skin. Here, we present a case of a nine-month-old male infant who presented with multiple noncompressible blue-purple subcutaneous nodules, initially suggestive of atypical deep hemangiomas. The ultrasound revealed a lack of increased vascularity of the masses and an adrenal mass, leading to a biopsy and diagnosis of a neuroblastoma involving the adrenal gland, liver, and skin. We demonstrate that metastatic neuroblastoma can mimic infantile hemangiomas and that noncompressible skin masses warrant further workup with ultrasonography to rule out neuroblastoma.

## Introduction

Neuroblastoma is a cancer of the peripheral sympathetic nervous system that forms from primordial neural crest cells [1]. Neuroblastoma is the most common extracranial cancer in children with an incidence of 600 new cases per year in the United States [1]. The White male population has a slightly higher incidence [1]. The median age of diagnosis is 22 months [1].

The adrenal glands are the most common original sites of neuroblastoma, followed by the paraspinal sympathetic ganglia [1]. Primary cutaneous neuroblastoma is rare [2]. Neuroblastoma may metastasize to other locations such as the liver and bones [1]. There are rare reports of metastasis to the skin [3]. Metastasis can present with fever, failure to thrive, bone pain, or blue subcutaneous nodules [1].

Neuroblastoma presenting as blue subcutaneous nodules can present a diagnostic challenge, as the lesions may present similarly to infantile hemangiomas. Infantile hemangiomas are benign vascular tumors that often appear within the first few months of life [4]. Infantile hemangiomas are common with a prevalence of approximately 5% among newborns [4]. They often grow over time and then spontaneously involute [4]. Hemangiomas can be superficial or deep [4]. Superficial infantile hemangiomas usually appear erythematous and well-defined and are compressible [4]. In contrast, the skin overlying the deep hemangiomas may appear skin-colored or blue, and the lesions are generally less defined but are still compressible [4]. As infantile hemangiomas are benign and involute spontaneously, treatment is usually not necessary [4].

## Case presentation

A nine-month-old White male patient presented to a pediatric dermatology clinic with multiple non-tender subcutaneous nodules on the face, chest, back, flank, and legs present since two weeks of age. Some of the nodules had grown slightly. A new lesion appeared two days before the presentation. He was referred to a pediatric dermatology with a presumptive diagnosis of multiple infantile hemangiomas.

**Figure 1:**
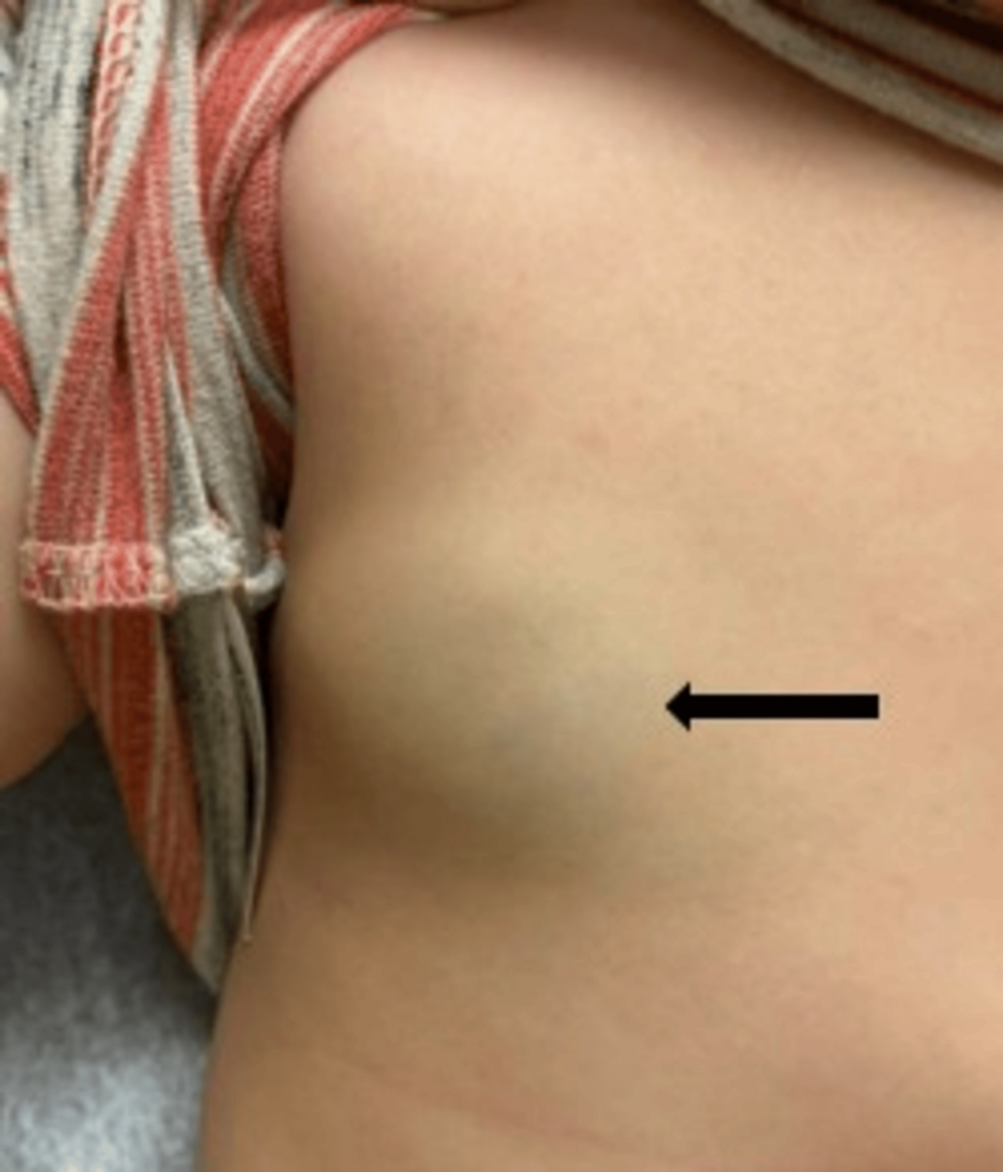
Firm, noncompressible blue-purple nodule on the anterior trunk (arrow).

On exam, the patient had multiple 1-4-cm firm, noncompressible, blue-purple nodules (Figure 1). The clinical impression was multiple deep infantile hemangiomas versus other tumors. With the presumptive diagnosis of infantile hemangiomas, propranolol therapy was initiated. Imaging was ordered for further evaluation given the atypical nature of the findings.

**Figure 2:**
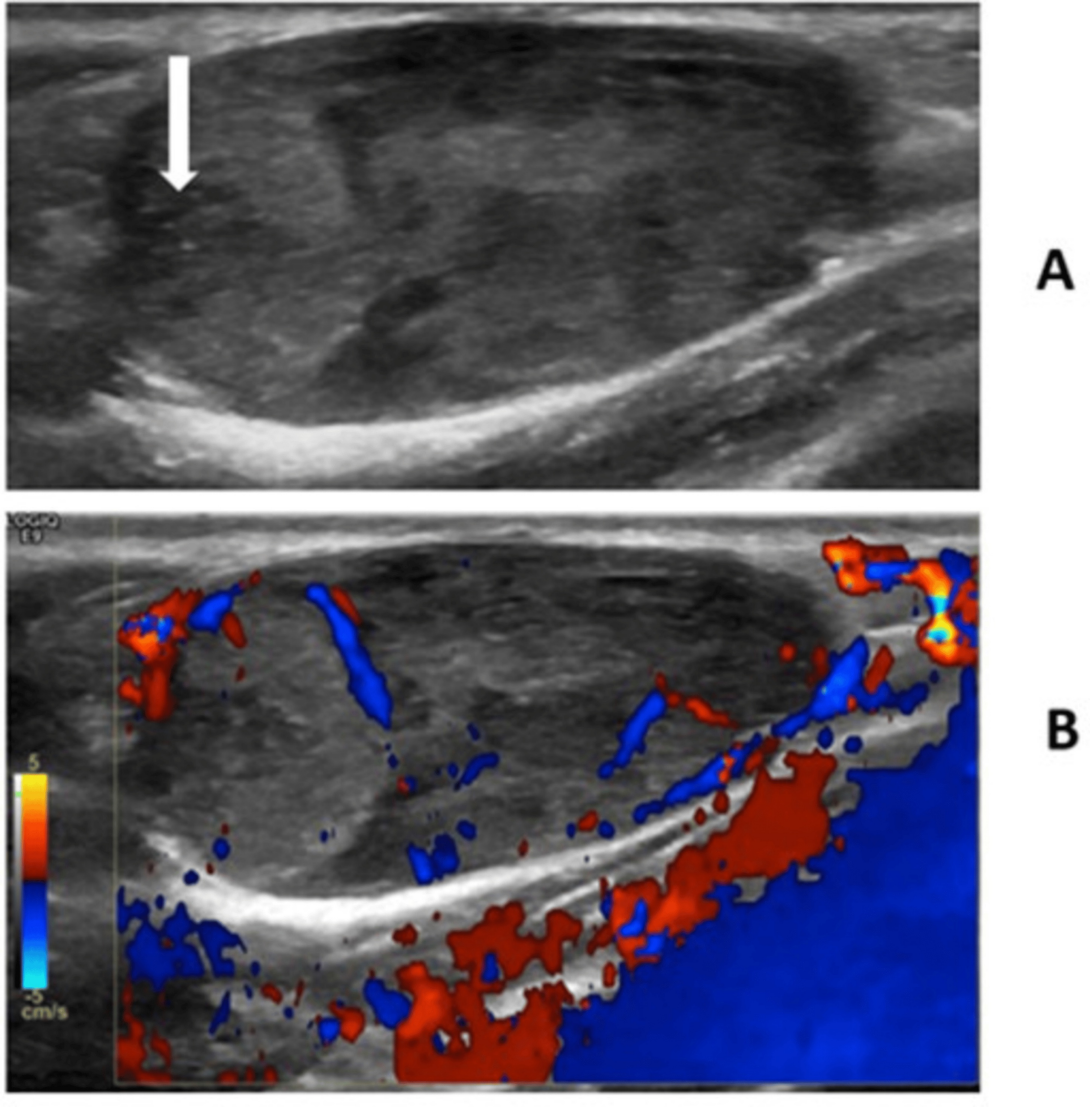
Grayscale (A) and Doppler (B) images of a lesion in the right upper back showing heterogenous echogenicities and regions of internal Doppler flow. There is a lack of diffuse increased vascularity typically seen in hemangiomas. There is questionable tiny calcification (arrow).

**Figure 3:**
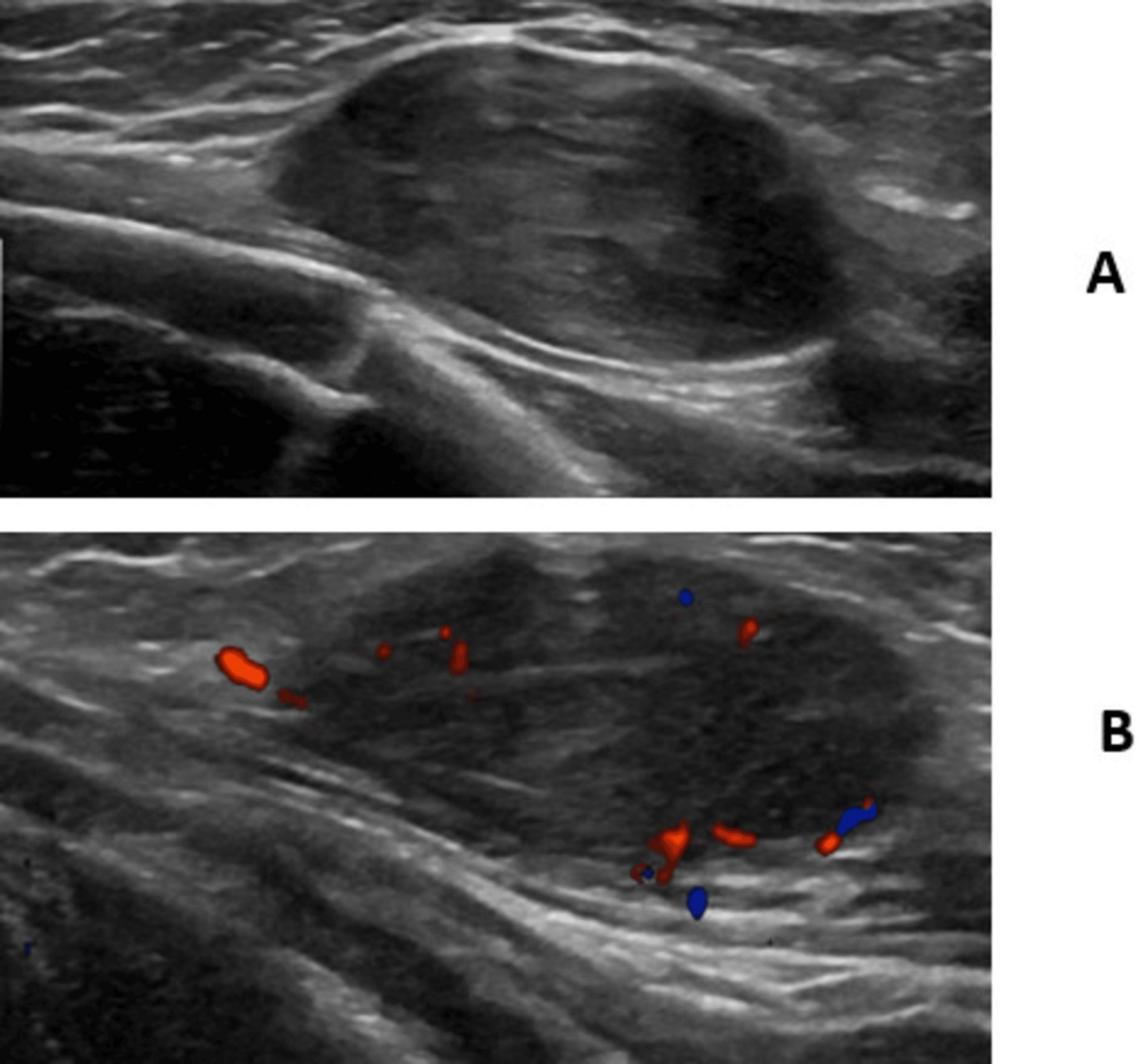
Grayscale (A) and Doppler (B) images of a palpable mass in the left anterior chest inferior to the clavicle. There is minimal vascularity.

**Figure 4:**
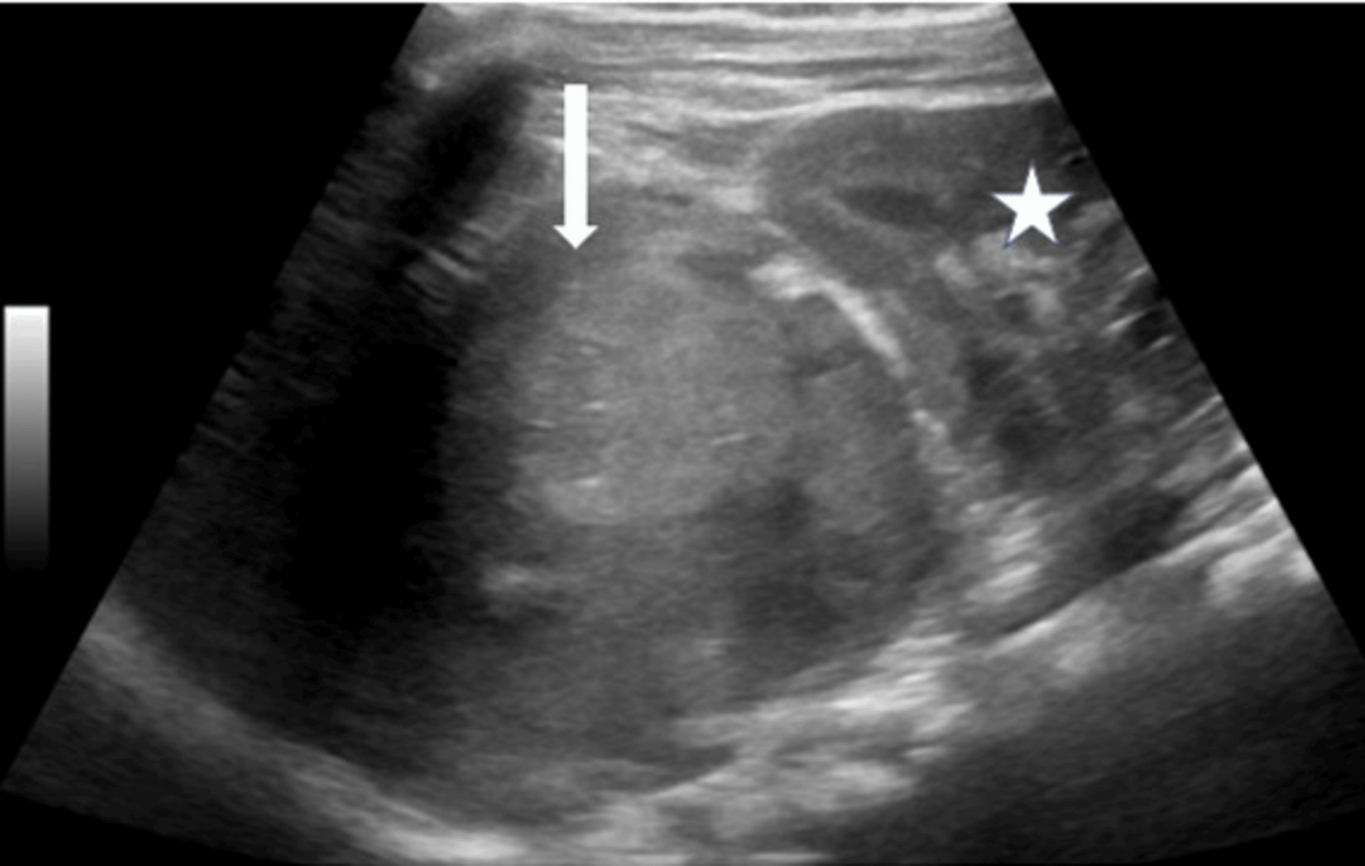
Grayscale image showing a large heterogenous mass in the left upper quadrant (solid arrow) with mass effect against the superior pole of the left kidney (star).

The ultrasound (US) revealed heterogeneities with coursing vessels and potential microcalcifications. The lesions did not have significant vascularity and were not predominantly hyperechoic (Figure 2, Figure 3). Thus, an abdominal US was performed, revealing a large heterogeneous mass with internal hyperechoic rounded regions and blood vessels in the left suprarenal area (Figure 4). The mass was separate from and displaced the kidney inferiorly. The liver was 8.2 cm with a focal hypoechoic region in the right lobe, an echogenic halo 9 x 7 x 4 mm in size, and questionable additional small hypoechoic foci. The MRI showed a circumscribed 6-cm adrenal mass, a sub-centimeter hepatic lesion, and numerous solid enhancing masses in the fats and muscles.

The imaging suggested a metastatic neuroblastoma. A subcutaneous mass biopsy showed stroma-poor neuroblastoma infiltrating the fibroadipose and fibroconnective tissues, confirming a diagnosis of neuroblastoma metastatic to the skin.

## Discussion

Our report adds to a limited but growing body of previous studies on metastatic neuroblastoma mimicking benign, infantile hemangiomas. One previous study reports on a lesion with a preliminary diagnosis of periorbital infantile hemangioma, but a noncompressible nature of the lesion prompted workup with MRI and biopsy, resulting in a final diagnosis of a neuroblastoma [5]. An additional case describes new deep skin-colored lesions appearing at 10 months of age, prompting further workup of presumed infantile hemangiomas [6]. Their US and biopsy histopathologies were consistent with neuroblastoma [6]. US is often the first step in assessing possible neuroblastoma [7]. Our report combined with previous reports demonstrates the utility of US in pursuing further workup of presumed infantile hemangiomas when atypical features are present. Our report demonstrates that atypical features include lesions that are noncompressible or appear after the first few months of a child’s life. Prompt further workup with US is necessary to allow for early recognition and treatment.

## Conclusions

In conclusion, we report a case of a neuroblastoma metastatic to the skin mimicking multiple infantile hemangiomas. We highlight the importance of US in evaluating an atypical presentation of presumptive infantile hemangiomas. In this case, new noncompressible lesions at nine months of age compelled us to investigate further with US. Clinicians should be aware of this rare presentation of metastatic neuroblastoma to aid in the early diagnosis.
